# Delta Opioid Receptor Agonists Ameliorate Colonic Inflammation by Modulating Immune Responses

**DOI:** 10.3389/fimmu.2021.730706

**Published:** 2021-09-22

**Authors:** Kazuki Nagata, Hiroshi Nagase, Ayumi Okuzumi, Chiharu Nishiyama

**Affiliations:** ^1^Laboratory of Molecular Biology and Immunology, Department of Biological Science and Technology, Faculty of Advanced Science and Technology, Tokyo University of Science, Tokyo, Japan; ^2^International Institute for Integrative Sleep Medicine (WPI-IIIS), University of Tsukuba, Tsukuba, Ibaraki, Japan; ^3^Global Science Campus, Tokyo University of Science, Tokyo, Japan

**Keywords:** DOR, IL-6, Inflammatory bowel disease, macrophage, opioid, Treg

## Abstract

The opioid receptors play important roles in the regulation of sense and emotions. Although it is recently revealed that opioid receptors are also expressed in various cells, but not restricted in the central nervous system, the effects of opioids on peripheral immune cells are largely unknown. In the current study, we evaluated the effect of opioids on immune system by using selective agonists for δ opioid receptor. Systemic administration of KNT-127 or intraperitoneal injection of YNT-2715 (a KNT-127-related compound that cannot pass through the blood-brain barrier) significantly alleviated the pathology of dextran sodium sulfate-induced colitis. In KNT-127-treated mice, the levels of an inflammatory cytokine IL-6 in the serum, and macrophages in the mesenteric lymph nodes (MLNs) were decreased in the progression stage, and those of regulatory T cells (Tregs) in the MLN were increased in the recovery stage. *In vitro* experiments revealed that KNT-127 inhibited the release of IL-6 and another inflammatory cytokine TNF-α from macrophages and accelerated the development of Tregs. Our study suggests that δ opioid agonists act directly on immune cells to improve the pathology of the colitis and can be candidates of immunomodulatory drugs.

## Introduction

Opioids are substances that control a variety of biological processes, such as pain, itch, emotions, and autonomous locomotion. Historically, a natural compound, morphine, which is the most famous opioid extracted from the poppy seed, was used as an anesthetic and analgesics even before its receptors and the endogenous opioid peptides were discovered. Opioids are classified into three types in terms of specific receptors: briefly, μ, κ and δ, bind to μ opioid receptor (MOR), κ opioid receptor (KOR), and δ opioid receptor (DOR), respectively, all of which are highly expressed in the central nervous system (CNS). The genes encoding opioid receptors and endogenous opioid peptides were identified in the 1990s (1–4), and the physiological roles of these molecules have been analyzed by using the gene-targeted mice, with research mainly focusing on the CNS (5–8). Stimulation of MOR induces the strongest analgesic effect and, in addition, has a large risk of serious side effects, such as dependence and respiratory depression (7). Although KOR signaling exhibits analgesic and antipruritic effects without causing dependence or respiratory problems, a certain kind of κ opioid often induces other kinds of aversion (9). On the other hand, DOR signaling has an advantage in that it does not induce apparent adverse reactions except convulsion and catalepsy, even though the analgesic activity of DOR is relatively low compared with that of MOR (8). Therefore, DOR agonists rather than MOR agonists have attracted attention as target analgesics.

In an analysis using mice with the *Oprd1* gene (encoding DOR) or the *Penk* gene (encoding preproenkephalin, a precursor peptide of an endogenous ligand for DOR called as enkephalin) knocked out, the physiological role of the δ opioid-DOR axis was revealed, mainly focusing on psychology and ethology. Based on previous studies demonstrating that *Penk* knockout (KO) mice show increased anxiety behavior and aggression (10), and *Oprd1* KO mice show increased depression-like behavior (8), DOR has been considered to be a promising target for antidepressants and anxiolytics in addition to analgesics. Subsequently, numerous agonists have been developed. KNT-127 is one of the most active and selective DOR agonists, as it has high permeability of the blood-brain barrier without inducing convulsions and catalepsy, which are caused by some other DOR agonists (11). Owing to the development of KNT-127, the physiology of DOR has been further analyzed, and it was revealed that DOR is involved in memory and feeding (12–14). However, the effects of KNT-127 on peripheral tissues other than the CNS have not been clarified.

The association between opioid receptors and the immune system has been suggested in previous studies on morphine (15–17). Epidemiological studies have revealed that patients treated with morphine are vulnerable to bacterial and viral infections and tend to have progression of some kinds of cancer (18–20). It was also reported that morphine and endogenous opioids modulate immune cell functions *in vitro* (15, 16, 21–24). Although these observations indicate that opioid receptors are involved in immune responses, the details for the roles of opioid receptors in immune regulation are largely unknown.

Here, we investigated how a δ opioid, KNT-127, affects the pathology of dextran sodium sulfate (DSS)-induced colitis and found that KNT-127 administration improved the colitis condition by regulating immune responses. The current study demonstrates that DOR agonists could be promising candidates of immunomodulatory drugs.

## Materials and Methods

### Mice

C57BL/6 mice were purchased from Japan SLC (Hamamatsu, Japan). Mice were maintained under specific pathogen-free conditions. All animal experiments were performed in accordance with the guidelines of the Institutional Review Board of Tokyo University of Science. The current study was specifically approved by the Animal Care and Use Committees of Tokyo University of Science: K21004, K20005, K19006, K18006, K17009, and K17012.

### Induction and Assessment of DSS-Induced Colitis

Female mice (6 weeks old) were given 2.5% (w/v) DSS, (#160110, MP Biomedicals, Santa Ana, USA) in their drinking water for 8 days. Mice were injected intraperitoneally (i.p. injection) with 5 mg/kg KNT-127 or YNT-2715, which was solubilized in saline, every 3 days beginning 3 days before DSS administration. Intracerebroventricular (i.c.v.) administration was performed with a microsyringe (#702LT, HAMILTON, Bonaduz, Switzerland) and an animal testing injection needle (two-step needle 2 mm, Natsume Seisakusho, Tokyo, Japan). Changes in weight were determined by weighing mice daily, and disease activity index (DAI) score (25) assessment was conducted every other day based on **Table 1**. In the recovery model, mice were maintained with 2.5% DSS water for 8 days and with normal water for an additional 4 days. KNT-127 or saline was i.p. injected into mice not treated with DSS every 3 days for 14 days to evaluate the effect of KNT-127 on leukocyte populations in the spleen. At sacrifice, we harvested the colon, spleen, mesenteric lymph nodes (MLNs), and blood to measure colon length, extract mRNA, collect leukocytes, and determine cytokine concentrations, respectively.

**Table 1:** Criteria of clinical index.

Score	Weight loss	Diarrhea	Blood in the stool
0	~ 1%	Hard	Negative
1	2 ~ 5%	Soft	Fecal occult blood
2	6 ~ 10%	Loose	Bloody stool (~ 49%)
3	10 ~ 15%	Muddy	Bloody stool (50% ~)
4	15% ~	Watery	Flesh blood

### Cells

Bone marrow-derived macrophages (BMDMs) were generated from bone marrow cells harvested from male mice as previously described (26). Briefly, hemolyzed whole-bone marrow cells were cultured in RPMI 1640 medium (#R8758, Sigma-Aldrich, St. Louis, USA) containing 10% FCS (#51820, Biowest, Nuaille, France), 100 U/mL penicillin, 100 μg/mL streptomycin, 100 μM 2-ME, 10 μM minimum nonessential amino acid solution, 100 mM sodium pyruvate, 10 mM HEPES and 20 ng/mL mM-CSF (#135-14391, Wako, Osaka, Japan) for 6 days. To stimulate BMDMs, 100 ng/mL lipopolysaccharide (LPS) (#L3024, Wako) was added to the culture supernatant. Naïve CD4^+^ T cells were isolated from the mouse spleen with the MojoSort Mouse Naïve CD4^+^ T Cell Isolation Kit (#480040, BioLegend, San Diego, USA) and were maintained in RPMI-based medium (the same medium as that used for BMDM generation but not containing M-CSF) in a plate coated with anti-CD3ϵ (clone; 145-2111C, BioLegend) and anti-CD28 (clone; 37.51, TONBO Bioscience) antibodies for 3 days. For the Treg-polarizing conditions, medium containing 3 ng/mL TGF-β1 (#763102, BioLegend) was used.

### Flow Cytometry

Whole splenocytes and MLN cells were stained with the following antibodies (Abs) to identify dendritic cells (DCs), macrophages, and regulatory T cells (Tregs): anti-CD11c-PE/Cyanine7 (clone; N418, TONBO Biosciences), anti-I-A/I-E-PerCP (clone; M5/114.15.2, BioLegend), anti-CD11b-APC-Cyanine7 (clone; M1/70, TONBO Bioscience), anti-F4/80-PE (clone; REA126, Miltenyi Biotec, Bergisch Gladbach, Germany), anti-CD3ϵ-PerCP (clone; 145-2C11, BioLegend), anti-CD4-FITC (clone; GK1.5, BioLegend), anti-CD64-PerCP/Cyanine5.5 (clone; X54-5/7.1, BioLegend), anti-Gr-1-FITC (clone; RB6-8C5, BioLegend), anti-CD19-PE/Cyanine7 (clone; 6D5, BioLegend). For Foxp3 staining, an anti-Foxp3-APC Ab (clone; 3G3, TONBO Bioscience) and the Foxp3/Transcription Factor Staining Buffer Kit (#TNB-0607-KIT, TONBO Biosciences) were used according to the manufacturer’s instructions. Intracellular staining was also performed to detect IFN-γ, IL-6, and IL-17A in CD4^+^ T cells using anti-IFN-γ-PE/Cyanine7 (clone; XMG1.2, BioLegend, anti-IL-6-APC (clone; MP5-20F3, BioLegend, and anti-IL-17A-APC/Cyanine7 (clone; TC11-18H10.1, BioLegend). To detect DOR, anti-Delta Opioid Receptor Ab (rabbit mAb #EPR5029 (2), abcam, USA) and FITC-conjugated Affinipure Goat Anti-Rabbit IgG(H+L) (#SA00003-2, Proteintech Group Inc., IL, USA) were used. Fluorescence was detected by a MACS Quant Analyzer (Miltenyi Biotec) and analyzed with Flowjo (Tomy Digital Biology, Tokyo, Japan).

### Quantitative RT-PCR

Total RNA was extracted from colon tissue and brain tissue using ISOGEN (#311-07361, Nippongene, Tokyo, Japan) and from BMDMs using the ReliaPrep RNA Cell Miniprep System (#Z6012, Promega, Madison, USA). After cDNA was synthesized from the isolated RNA by reverse transcription (RT) using ReverTra Ace qPCR RT Master Mix (#FSQ-201, TOYOBO, Osaka, Japan), real-time quantitative PCR was performed with Thunderbird SYBR qPCR Mix (#QPS-201, TOYOBO) on the StepOne Real-Time PCR System (Applied Biosystems, Kanagawa, Japan). The nucleotide sequences of the primer sets used to detect specific genes are shown in **Table 2**.

**Table 2:** Nucleotide sequences of primers used in quantitative PCR.

gene	Forward	Reverse
*Defa*	5’-ggctgtgtctgtctcctttgg-3’	5’-gatctctcgacgatttttcatgaa-3’
*Lypd8*	5’-tggctggacccagaaggat-3’	5’-tcaggacctggctagcagaca-3’
*Reg3b*	5’-cagaactggcctgccaaaa-3’	5’-gcgctattgagcacagatacga-3’
*Cdh1*	5’-tcgccctgctgattctgatc-3’	5’-ggctctttgaccaccgttctc-3’
*Cldnd1*	5’-ccgtagcatcttggagcagtct-3’	5’-caatcacaaacgcagtagcaaaa-3’
*Ocel1*	5’-ctcctgcaggctctccacat-3’	5’-tccctgcttcctgcagatg-3’
*Tgfb1*	5’-gctgaaccaaggagacggaat-3’	5’-tttgctgtcacaagagcagtga-3’
*Vegfa*	5’-aagccagaaaaaaaatcagttcga-3’	5’-gggatttcttgcgctttcg-3’
*Vegfd*	5’-agaccccagaagaagatgaatgtc-3’	5’-atcccacagcatgtcaatagga-3’
*Tnf*	5’-agggatgagaagttcccaaatg-3’	5’-tgtgagggtctgggccata-3’
*Il6*	5’-aatcgtggaaatgagaaaagagttg-3’	5’-agtgcatcatcgttgttcatacaa-3’
*Oprm*	5’-ggtctgcccgtaatgttcatg-3’	5’-aacgtgagggtgcaatctatg-3’
*Oprk*	5’-atagtccttggaggcaccaaa-3’	5’-ggaaactgcaaggagcattca-3’
*Oprd*	5’-gggcttctgggcaacgt-3’	5’-ggcggtcttcaatttggtgta-3’
*Pomc*	5’-ctcaccacggagagcaacct-3’	5’-tcatctccgttgccaggaa-3’
*Pdyn*	5’-gccaccctaccacctgatca-3’	5’-caacgcctctgcttactgctt-3’
*Penk*	5’-tgcagctaccgcctggtt-3’	5’-cagtgtgcacgccaggaa-3’
*Gapdh*	5’-acgtgccgcctggagaa-3’	5’-gatgcctgcttcaccacctt-3’

### ELISA

The concentrations of TNF-α and IL-6 were determined with ELISA MAX Deluxe Set Mouse kits (#430904 for TNF-α, #4313004 for IL-6, BioLegend). A mouse C-Reactive Protein/CRP Quantikine ELISA kit (#MCRP00, R&D Systems Inc., MN, USA) was used to measure the serum CRP concentration.

### Statistical Analysis

**Figure 1:**
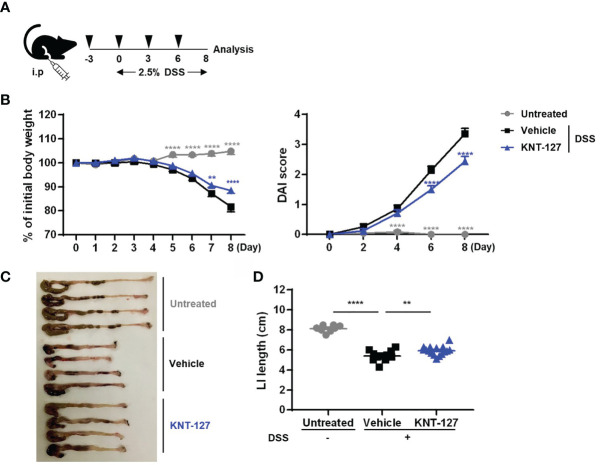
Effects of KNT-127 on DSS-induced colitis. **(A)** Mice were given water containing 2.5% DSS to induce colitis starting on day 0, and 5 mg/kg KNT-127 (n=16) or vehicle (n=12) was administered intraperitoneally to the mice every 3 days from day -3 to day 6. Control mice (n=8) were given normal water. **(B)** The percentage of body weight to initial one (left). Disease activity index (DAI) scores (right). **(C)** Images of the colon and cecum on day 8. **(D)** Length of the large intestine. Symbol key: ○, control; □, vehicle; and △, KNT-127. The data are shown as the mean ± s.e.m. **P < 0.01; ****P < 0.0001 (one-way ANOVA followed by Dunnett’s multiple comparisons test, *vs* vehicle). i.p., intraperitoneal.

**Figure 2:**
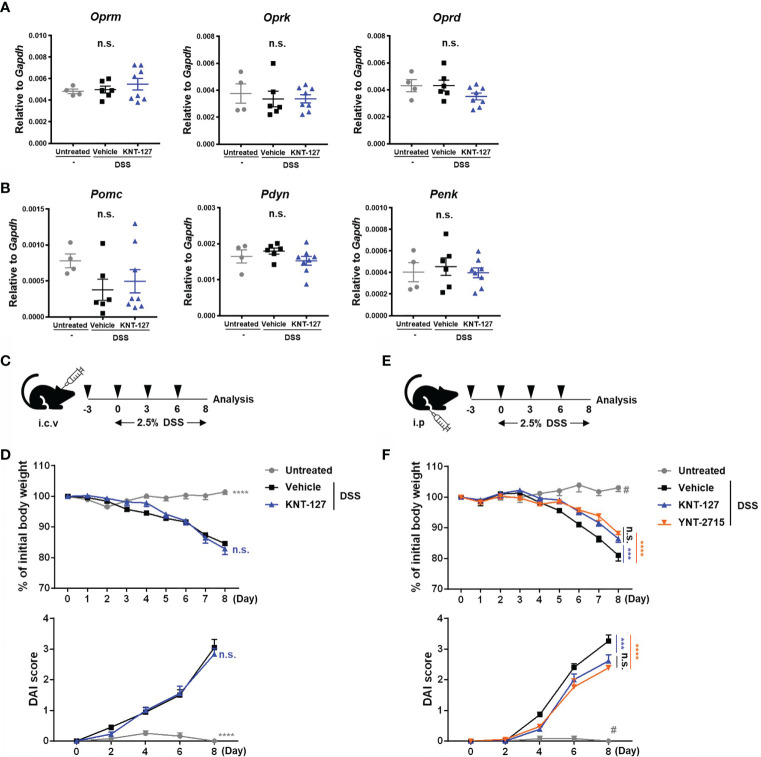
Identification of the site of KNT-127 activity. The whole brain was collected from colitis mice. The mRNA expression of each gene of interest was normalized to that of GAPDH by calculation of cycle threshold values. **(A)** mRNA levels of opioid receptors. **(B)** mRNA levels of endogenous opioid precursors. **(C)** KNT-127 (5 μg/mouse, n=6) or vehicle (n=6) was administered intraventricularly to mice every 3 days from day -3 to day 6. **(D)** The percentage of body weight (top). DAI scores (bottom). **(E)** KNT-127 (5 mg/kg, n=8), YNT-2715 (5 mg/kg, n=8) or vehicle (n=6) was administered intraperitoneally to mice every 3 days from day -3 to day 6. **(F)** The percentage of body weight (top). DAI scores (bottom). ○, control; □, vehicle; △, KNT-127; and ▽, YNT-2715 respectively. The data are shown as the mean ± s.e.m. ***P < 0.001; ****P < 0.0001; n.s., not significant (one-way ANOVA followed by Dunnett’s multiple comparisons test, *vs* vehicle). ^#^ *P* < 0.05 (one-way ANOVA followed by Turkey’s multiple comparison test, Untreated vs all other groups). i.c.v., intracerebroventricular; i.p., intraperitoneal.

A two-tailed Student’s t-test was used to compare two samples (**Figures 4D, F**, **5B**, **Supplementary Figure 3**) and one-way ANOVA followed by Dunnett’s multiple comparisons test was employed to compare multiple samples to the corresponding control (**Figures 1** **–** **3**, **Figures 4A–C, E**, **6B, D**, **Supplementary Figure 1**, **Supplementary Figure 2**, and **Supplementary Figure 5**). We used Turkey’s multiple comparison test in **Figure 2F**. *P* values < 0.05 were considered significant.

**Figure 3:**
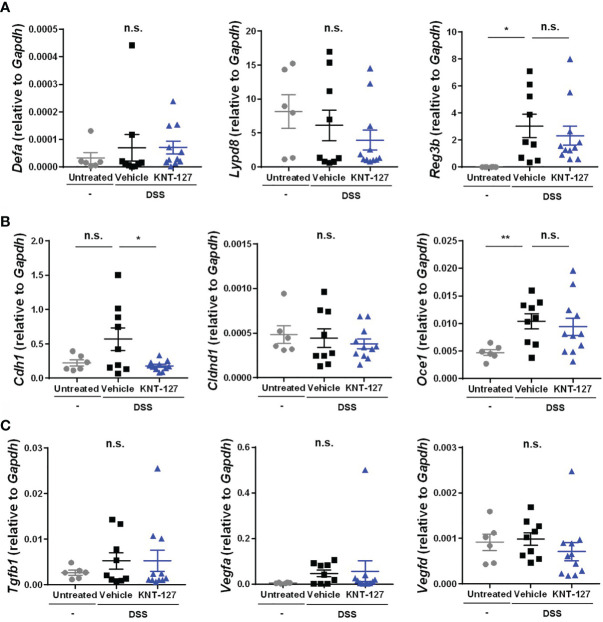
Changes in the intestinal factors. The colon was collected from colitis mice. The mRNA expression of each gene of interest was normalized to that of GAPDH by calculation of the cycle threshold values. **(A)** mRNA levels of antimicrobial peptides. **(B)** mRNA levels of tight junction proteins. **(C)** mRNA levels of growth factors. The data are shown as the mean ± s.e.m. *P < 0.05; **P < 0.01; n.s., not significant (one-way ANOVA followed by Dunnett’s multiple comparisons test, *vs* vehicle).

**Figure 4:**
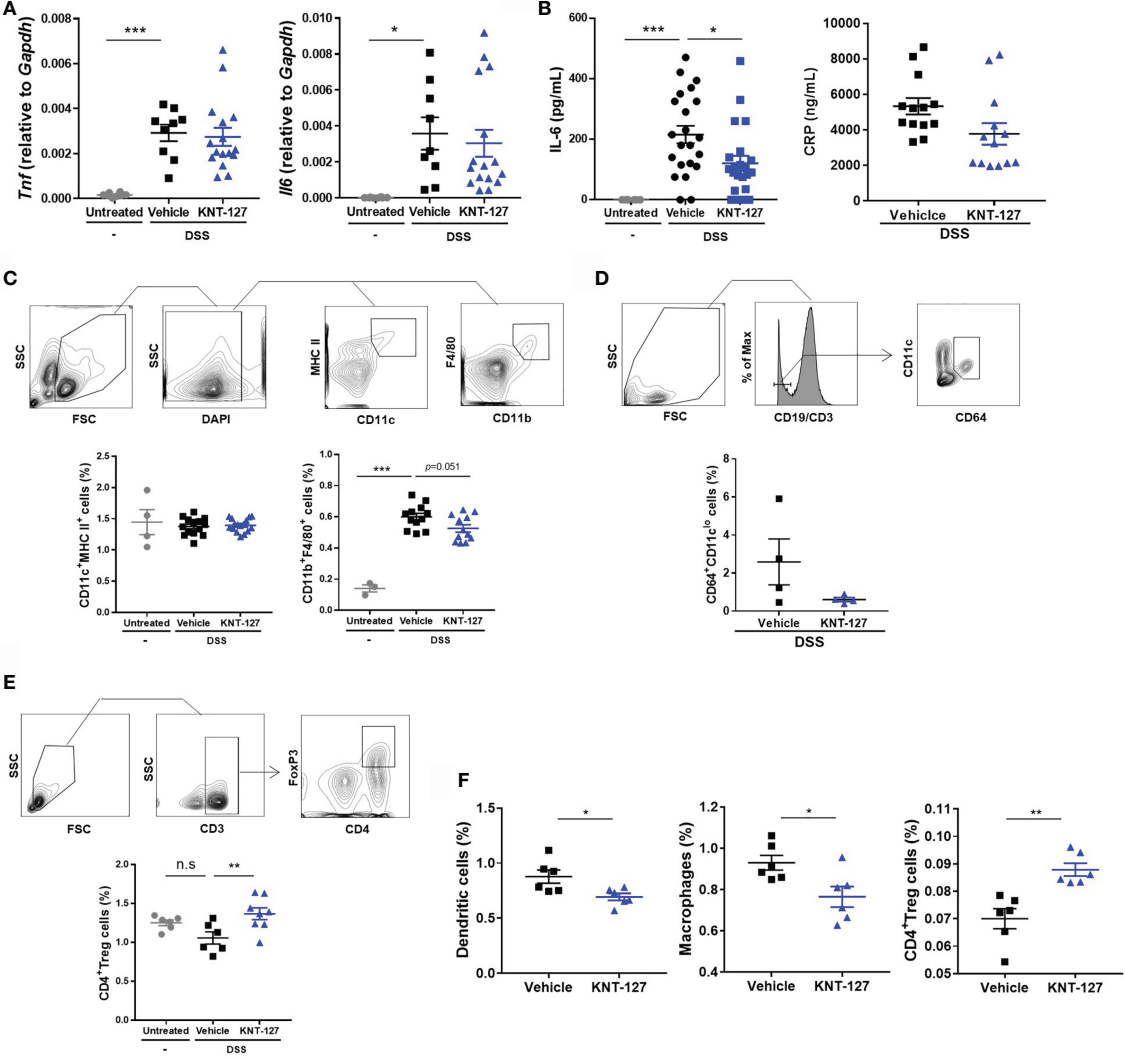
Changes in the immune system. **(A)** mRNA levels of *Tnf* and *Il6.* The mRNA expression of each gene of interest was normalized to that of GAPDH by calculation of cycle threshold values. **(B)** Serum was collected from colitis mice, and the concentrations of IL-6 and CRP were measured by ELISA. **(C)** The population of macrophages (F4/80^+^CD11b^+^ cells) in the MLNs was evaluated by flow cytometry. **(D)** The frequency of CD64^+^/CD11c^lo^ cells in the MLNs of colitis mice. **(E)** The population of Tregs (CD3ϵ^+^CD4^+^Foxp3^+^ cells) in the MLNs was evaluated by flow cytometry. **(F)** The populations of DCs (CD11c^+^MHCII^+^ cells), macrophages, and Tregs in the spleen were evaluated by flow cytometry. The data are shown as the mean ± s.e.m. *P < 0.05; **P < 0.01; ***P < 0.001; n.s., not significant (one-way ANOVA followed by Dunnett’s multiple comparisons test for **A–C, E**, and two-tailed Student’s t-test for **D, F**).

## Results

### Systemic Administration of the δ Opioid Receptor Agonist KNT-127 Alleviates the Pathology of DSS-Induced Colitis

We first verified the effect of systemic administration (i.p. injection) of the DOR agonist KNT-127 on inflammatory bowel disease (IBD) using mouse colitis models (**Figure 1A**). The pathology of DSS-induced colitis is characterized by monitoring body weight; the length of the colon, which reflects the fibrosis level; and DAI scores, which are calculated from the degrees of weight loss, diarrhea, and blood in the stool. As a result of analyzing weight changes over 9 days, we found that the DSS-induced weight loss was slightly but significantly relieved in mice treated with KNT-127 (**Figure 1B** left). In addition, the DAI score, indicating the severity of colitis, was apparently improved by KNT-127 administration (**Figure 1B** right). Furthermore, measurement of colon length at sacrifice (day 8) revealed that the atrophy caused by DSS was reduced in KNT-127-treated mice (**Figures 1C, D**). The beneficial effect of KNT-127 was also observed in a colitis recovery model (**Supplementary Figures 1A, B**). These results suggest that DOR signaling is involved in the pathology of IBD in mice.

### Effects of Systemic Administration of a DOR Agonist on the Expression of Endogenous Opioids and Their Receptors in the CNS

In many cases, opioid receptor signaling has been reported to affect systemic physiology, mainly through the CNS (6, 27, 28). Thus, we wondered whether the effect of KNT-127 on DSS-induced colitis is caused by modulation of the CNS. First, to clarify this point, we examined the possibility that DSS and/or KNT-127 treatment alters the expression levels of endogenous opioids and their receptors in the brain of colitis mice and its control mice. As shown in **Figures 2A, B**, we determined the mRNA levels of the opioid receptors *Oprm* (MOR), *Oprk* (KOR), and *Oprd* (DOR) and their ligand precursors, including *Pomc* (Pro-opiomelanocortin), *Pdyn* (Prodynorphin) and *Penk* (Proenkephalin) in the whole brain, which is the main source of endogenous opioids and plays an important role in the opioid system. Quantitative PCR showed that neither DSS treatment nor KNT-127 administration caused significant changes in the mRNA levels of the receptors (**Figure 2A**) or agonist precursors (**Figure 2B**) in the brain.

### DOR Signaling in the CNS Is Not Involved in the DOR Agonist-Mediated Alleviation of Bowel Inflammation

Next, to further explore the involvement of the CNS in the KNT-127-mediated improvement in bowel inflammation, we investigated the effects of local injection of different types of DOR agonists: KNT-127 and YNT-2715 (**Figures 2C, E**). It was confirmed that KNT-127 administered intraventricularly at a dose of 5 μg/mouse did not diffuse out from the brain, whereas KNT-127 i.p. injected at a dose of 5 mg/kg spread throughout the whole body (11). Then, we performed i.c.v. infusion of KNT-127 to evaluate the effect of CNS-restricted administration of a DOR agonist on colitis pathology. As shown in **Figure 2D**, both the weight loss and DAI score in DSS-induced colitis were not affected by i.c.v infusion of KNT-127, suggesting that stimulation of DOR signaling in the CNS is not involved in the improvement in bowel inflammation in the mouse model. In the next experiment, we used YNT-2715, the second DOR agonist investigated in the current study, which cannot pass through the blood-brain barrier, unlike KNT-127, because of the relatively low hydrophobicity of YNT-2715, which carries methyl iodide (29, 30). The DSS-induced decrease in body weight and increase in the DAI score were significantly alleviated by i.p. administration of KNT-127, as observed in **Figure 1** (blue triangles in **Figure 2F**). In this experimental condition, we found that the i.p. administration of YNT-2715, which is expected to diffuse into the CNS at only very low levels, effectively recovered the weight loss and DAI score (red inverted triangles in **Figure 2F**), as observed with i.p. injection of KNT-127. Furthermore, we found that pre-treatment of colitis mice with a DOR antagonist just before injection of KNT-127 suppressed the recovery degree, suggesting that KNT-127 causes the improvement of colitis in the DOR-dependent manner (data not shown). These results demonstrate that the DOR signaling in the CNS is not required for the DOR agonist-mediated improvement in DSS-induced colitis.

### Expression Profiles of Genes Involved in Antibacterial and Barrier Functions in the Colon

The abovementioned results indicate the possibility that DOR agonists suppress bowel inflammation by affecting gene expression and/or cell function in peripheral tissues rather than modulating CNS-dependent regulation. To elucidate the mechanism by which DOR agonists relieve disease, we examined the gene expression profile of lesional colon tissue from the DSS-induced colitis mice. It has been reported that antimicrobial peptides, tight junction-related molecules, and growth factors expressed in colonic epithelial cells contribute to defense against colitis by inhibiting the infiltration of intestinal bacteria from the lumen (31–36). When we determined the mRNA levels of antimicrobial peptides in colon tissue, we found that the mRNA level of the inflammation-induced antimicrobial peptide *Reg3b* was increased by DSS administration but not affected by the additional administration of KNT-127, and that the mRNA levels of the constitutive peptides *Derfa* (α-defensin) and *Lypd8* (Lypd8) were comparable among the three groups (**Figure 3A**), suggesting that KNT-127 did not enhance the release of these antimicrobial peptides. Similarly, the mRNA levels of the tight junction components *Cldnd1* (Claudin domain containing 1) and *Ocel1* (Occludin/ELL domain containing 1), and those of the growth factors *Tgfb1* (TGF-β1), *Vegfa* (VEGF), and *Vegfd* (FIGF) were not increased by KNT-127 treatment (**Figures 3B, C**). Although a significant change in the expression of *Cdh1* (E-cadherin) was caused by KNT-127, considering that E-cadherin has a protective role in colitis (32), the decrease in *Cdh1* mRNA expression observed in KNT-127-treated mice was probably not the cause of the relief but reflected the reduction in inflammation (**Figures 3A–C**). Moreover, we confirmed that KNT-127 treatment did not cause significant changes in the expression of these molecules in the colitis recovery model (**Supplementary Figures 2A–C**) and that KNT-127 administration to healthy mice did not affect the expression of these molecules (**Supplementary Figures 3A–C**). These data indicate that KNT-127 exerts a beneficial effect on colitis through cells other than colonic epithelial cells.

### Effects of KNT-127 Treatment on the Immune System *In Vivo*

Since immune system-mediated inflammatory responses play key roles in colitis, we next analyzed the expression of inflammatory cytokines and populations of leukocytes in mice. The inflammatory cytokines TNF-α and IL-6 are well-known therapeutic targets in IBD that contribute to the deterioration of the colitis condition (37). Therefore, we first determined the mRNA levels of *Tnf* (TNF-α) and *Il6* (IL-6) in colon tissue. As expected, the mRNA levels of *Tnf* and *Il6* were markedly increased in colitis mice (**Figure 4A**). Although KNT-127 did not exhibit significant effects on the mRNA expression of *Tnf*, *Il6* mRNA levels tended to decrease in KNT-127-treated mice (**Figure 4A**). Accordingly, we measured the concentration of the IL-6 protein in serum, and found that the serum IL-6 concentration was obviously increased with the onset of the colitis and then significantly suppressed by KNT-127 administration (**Figure 4B**). In addition, we found that the serum concentration of CRP, which is synthesized in the liver upon IL-6 stimulation and is known as a common clinical parameter, tended to be suppressed by KNT-127-treatment (**Figure 4B**). Although the effect of KNT-127 was not significant in the recovery model, similar tendency was observed in the serum IL-6 concentrations, and mRNA levels of cytokines in the colon (**Supplementary Figures 1C, D**). To further clarify the mechanism by which serum IL-6 levels were downregulated in KNT-127-treated mice, we evaluated the populations of macrophages and DCs, which are the major sources of IL-6 (38, 39), in lymphoid organs by using flow cytometry. Although KNT-127 treatment did not affect the populations of macrophages or DCs in the spleen (data not shown) and DCs in the MLNs of colitis mice (**Figure 4C**), the macrophage population in the MLNs was apparently increased by DSS treatment and then tended to decrease in response to additional treatment with KNT-127 (**Figure 4C**). It is reported that the composition of immune cells drastically changed in the intestine under inflammatory conditions and CD64^+^/CD11c^lo^ is a useful marker to distinguish inflammatory macrophages from other monocytes (40). Therefore, we performed a flow cytometry using anti-CD64 Ab and found that the numbers of CD64^+^/CD11c^lo^ cells in the MLNs of colitis mice were decreased by KNT-127 treatment (**Figure 4D**). This may suggest that KNT-127 regulates the migration of activated macrophages. When we analyzed the leukocyte population in the MLNs in the colitis recovery model, we found that the frequency of Tregs was slightly decreased in DSS-treated mice and significantly increased in KNT-127-treated mice (**Figure 4E**), whereas KNT-127 treatment did not affect the frequency of other CD4^+^ T cell subtypes (**Supplementary Figure 4**). Considering that macrophages and Tregs in the MLNs are strongly associated with the degree of inflammation in the colon (41, 42), these results indicate that KNT-127 suppresses macrophage-mediated inflammation in the progression stage of colitis and enhances the Treg-mediated anti-inflammatory response in the recovery stage.

To examine whether KNT-127 modulates the localization of immune cells, we administered KNT-127 to mice not treated with DSS. As shown in **Figure 4F**, KNT-127 administration remarkably affected the populations of DCs, macrophages, and Tregs in the spleen. Briefly, the frequencies of DCs and macrophages were significantly decreased by KNT-127 treatment; conversely, that of Tregs was significantly increased (**Figure 4F**). These results suggest that systemic administration of KNT-127 affects the localization and/or development of immune cells.

**Figure 5:**
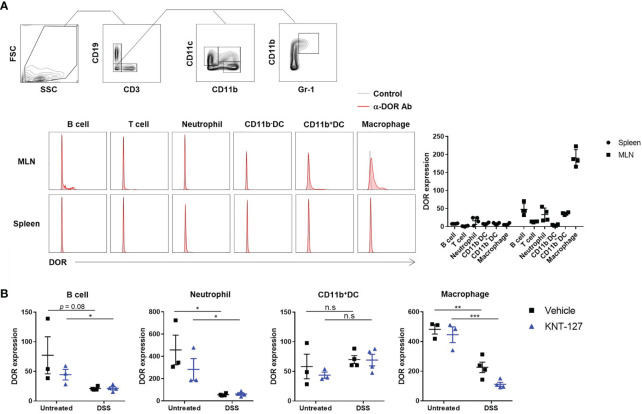
DOR expression levels on immune cells. **(A)** Cells prepared from the MLNs and spleen of normal mice. Gating strategies (top), typical histograms (bottom left), and MFIs (bottom right, n=4). Control, FITC-labeled anti-rabbit 2nd Ab only; α-DOR Ab, anti-DOR rabbit Ab plus 2nd Ab. **(B)** Effects of DSS treatment and/or KNT-127 treatment on DOR expression levels on immune cells in the MLNs. The data are shown as the mean ± s.e.m. *P < 0.05; **P < 0.01; ***P < 0.001; n.s., not significant (two-tailed Student’s t-test).

Above-mentioned results suggest that immune cells in the intestine directly responded to KNT-127. To clarify whether these cells express DOR on their surface to receive the signaling from KNT-127, we performed flow cytometry using anti-DOR Ab. As shown in **Figure 5A**, although the expression levels of DOR on hematopoietic cells isolated from the spleen were low, apparent expression of DOR was detected on immune cells from the MLNs, especially macrophages, CD11b^+^ DCs, neutrophils, and B cells. The expression levels of DOR on macrophages, neutrophils, and B cells in MLNs of colitis mice were lower than those of non-DSS-treated mice, whereas the DOR expression levels on DCs were not decreased by DSS-treatment (**Figure 5B**). Although the mechanism by which DSS treatment suppressed the expression of DOR on several immune cells is not revealed, considering that KNT-127 treatment further reduced the expression level of DOR on macrophages of colitis mice, the reduced expression levels of DOR on macrophages of colitis mice may be enough to respond to KNT-127.

### KNT-127 Suppresses Cytokine Release by Macrophages and Accelerates Treg Development *In Vitro*

**Figure 6:**
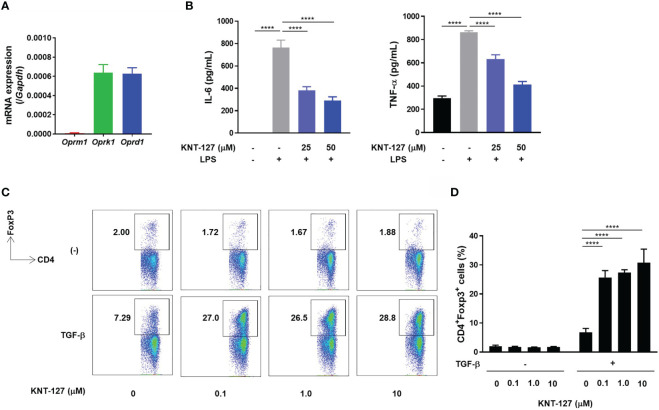
Direct effects of KNT-127 on immune cells *in vitro.* **(A)** mRNA levels of opioid receptors in BMDMs. The mRNA expression of each gene of interest was normalized to that of GAPDH by calculation of the cycle threshold values. **(B)** TNF-α and IL-6 protein concentrations in the culture supernatant of BMDMs. BMDMs (5.0×10^5^ cells/500 μL) were incubated in the presence of KNT-127 for 30 minutes, followed by stimulation with 100 ng/mL LPS. The culture supernatant was harvested 6 hours after LPS stimulation, and the TNF-α and IL-6 concentrations were measured by ELISA. **(C, D)** Naïve CD4^+^ T cells purified from the spleen were stimulated in a plate coated with anti-CD3ϵ and anti-CD28 Abs in the presence of 3 ng/mL TGF-β. The frequency of Tregs (CD3ϵ^+^CD4^+^Foxp3^+^ cells) was determined by flow cytometry. The data represent the mean ± SD of triplicate samples. Similar results were obtained in three independent experiments. ****P < 0.0001; n.s., not significant (one-way ANOVA followed by Dunnett’s multiple comparisons test, *vs* vehicle).

Finally, we performed *in vitro* experiments to clarify the direct effects of KNT-127 on immune cells. To evaluate the effect of KNT-127 on macrophages, we generated BMDMs. RT-PCR confirmed that BMDMs expressed apparent levels of mRNA transcripts encoding KOR and DOR (**Figure 6A**). Then, we determined the amounts of TNF-α and IL-6 secreted from LPS-stimulated BMDMs treated with KNT-127 at the indicated concentration. As shown in **Figure 6B**, KNT-127 suppressed the LPS-induced production of TNF-α and IL-6 by BMDMs in a dose-dependent manner.

We also investigated the effect of KNT-127 on the development of Tregs by monitoring the frequency of Foxp3^+^ cells in a CD4^+^ T cell population. When naïve CD4^+^ T cells isolated from the mouse spleen were cultured in TGF-β-supplemented medium, the population of Tregs characterized as Foxp3^+^/CD4^+^ was increased on day 3 (**Figure 6C**). Under this experimental condition, the addition of KNT-127 to the culture medium dramatically increased the frequency of Tregs, whereas the promotive effect of KNT-127 was not observed under the nonpolarizing condition (**Figures 6C, D**). This observation suggests that KNT-127 can strongly promote the differentiation of naïve CD4^+^ T cells into Tregs under polarizing conditions. Overall, KNT-127 directly regulates the function of immune cells, briefly suppresses the release of inflammatory cytokines by macrophages and accelerates the development of Tregs, which could be the probable mechanism of colitis relief.

## Discussion

**Figure 7:**
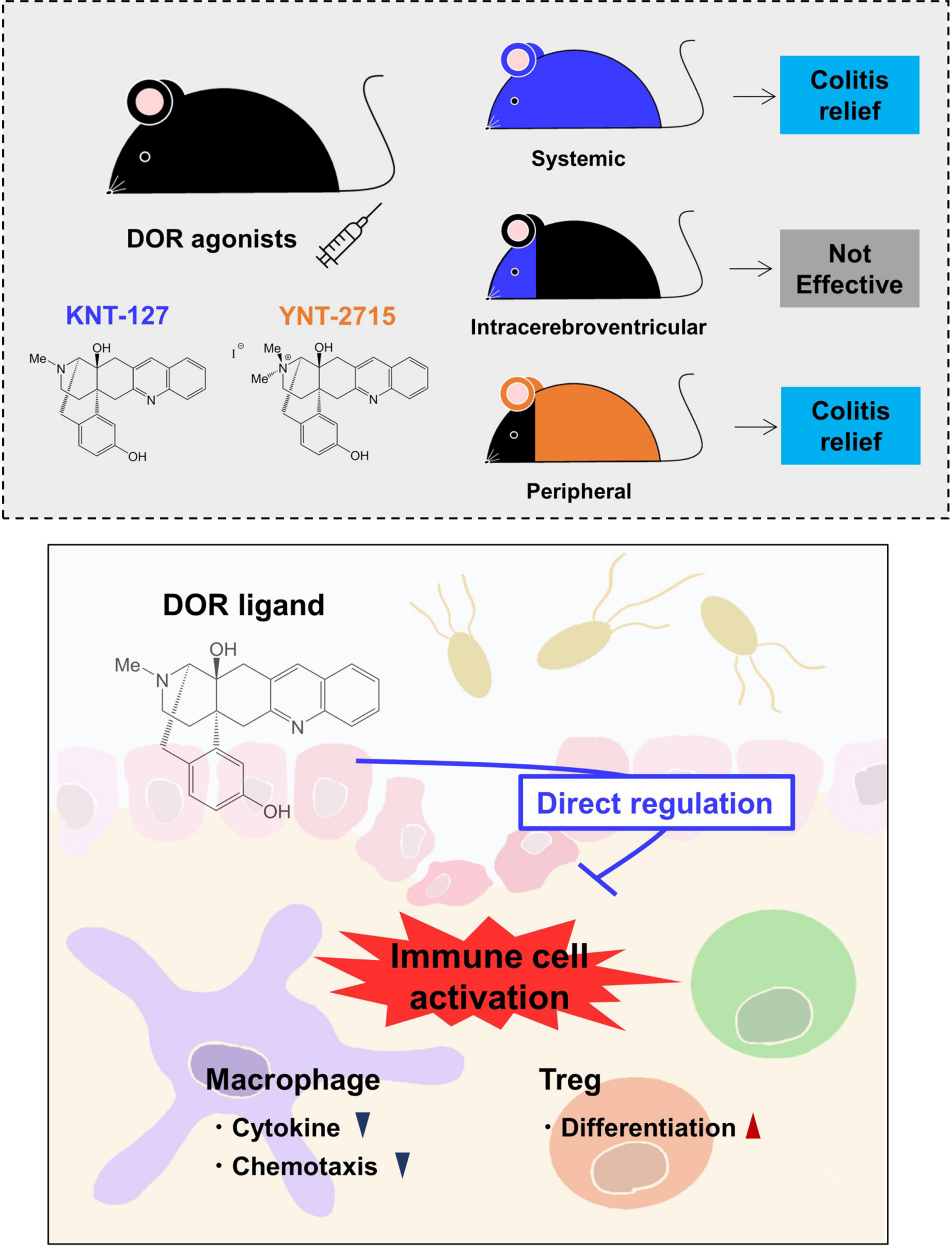
Schematic drawing of the effects of DOR agonists on immune responses. Systemic administration of a selective DOR agonist KNT-127 and peripheral (other than the CNS) administration of a DOR agonist YNT-2715 ameliorated colitis, but intraventricular administration of KNT-127 did not cause an apparent effect on colitis (top). KNT-127 suppressed the inflammatory responses of macrophages, and increased Treg numbers *in vivo* and *in vitro* (bottom).

The involvement of opioids in immune responses has been suggested by epidemiological studies and *in vitro* analyses of morphine (15, 23). However, the molecular mechanism by which opioids affect immune-related disorders is largely unknown, mainly due to the problem related to the receptor type selectivity of opioids. For example, morphine, which mainly targets MOR, also cross reacts with DOR. In the current study, we used KNT-127, which was developed as a DOR selective agonist (11). The systemic administration of KNT-127 *via* i.p. injection significantly suppressed DSS-induced colitis, whereas mice intraventricularly injected with KNT-127 did not show any improvement in colitis. In contrast, i.p. injection of the peripheral δ opioid YNT-2715, which cannot pass through the blood-brain barrier, reduced the pathology of colitis, suggesting that the DOR agonists ameliorate colitis without modulating the CNS (**Figure 7** top). We also found that the induction of colitis and treatment with a DOR agonist did not affect the expression levels of opioid receptors or endogenous opioids in the CNS, which may support the noninvolvement of the CNS in the effect of KNT-127 on the colitis. Determination of the expression levels of opioid peptide-processing enzymes and activated opioid peptides is required to further exclude the possible involvement of the CNS in colitis relief (43). Although KNT-127 possesses antianxiety, antidepressant, and analgesic effects (11–13), considering that intraventricular administration of KNT-127 did not cause an apparent effect on colitis, the regulation of behavior and psychology by KNT-127 was not the main cause of the observed colitis improvement; δ opioids improved colitis by acting on peripheral tissues and/or cells in the current study.

IBD is a condition in which inflammation develops in the gastrointestinal tract, which is associated with sores and ulcers, and has two distinct types; Crohn’s disease and ulcerative colitis (44). DSS-induced colitis is a model of ulcerative colitis and is generally used to elucidate the pathology of IBD and evaluate therapeutics (25). Regarding the pathology of DSS-induced colitis, it has been shown that the immune system is a participant, that is, in most cases, the immune response is involved in the exacerbation of colitis-inducing inflammatory cytokines and various chemokines (37, 45, 46).

Recently, a study analyzing the colonic visceromotor response of full or primary afferent neuron-specific KO of *Oprd* or *Oprk* was reported (47). Although *Oprd* ^-/-^ mice showed an increased visceromotor response to colorectal distension (CRD) under normal conditions, neither full KO nor conditional KO (cKO) of DOR affected the expression levels of opioid-related molecules and cytokines in the distal colon or aggravated DSS-induced colon hypersensitivity, as shown by the CRD index. The observation that whole-body deletion of DOR did not cause apparent effects on the expression of opioid receptors or their endogenous ligands in the colon (47) may be consistent with our result that the expression of opioid-related genes in the CNS was not affected by a systemic administration of KNT-127. Furthermore, the mRNA levels of antimicrobial peptides, growth factors, and tight junction-related molecules, which are known to participate in the pathology of colitis (31–36) were comparable between the vehicle group and KNT-127-treated group, which may also be consistent with DOR deletion no affecting DSS-induced colon hypersensitivity. Considering that DOR expression levels on several immune cells in MLNs were reduced in colitis mice, and that of macrophages was further decreased by KNT-127-treatment, detailed analyses including determination of the protein levels in isolated cells rather than mRNA levels in whole tissues may be required to evaluate the effect of DOR-signaling and/or DSS-treatment on the expression of endogenous opioids and opioid receptors in the CNS and in the colon.

A certain amount of DORs is expressed in the intestinal tract, especially in the nerve fibers of the mucosal lamia propria and muscle layer, which are involved in the inhibition of mucus secretion and intestinal peristalsis (48, 49). Nevertheless, considering that the activation of DOR in the intestinal tract suppresses intestinal peristalsis and reduces mucus secretion, KNT-127 in the intestine may cause a negative effect on the treatment of colitis. Briefly, there is a possibility that KNT-127 administration will elongate the retention of DSS in the intestinal tract, resulting in exacerbation of colitis rather than improvement due to an increase in the vulnerability of intestinal barriers, which will reduce the total effect of KNT-127 on colitis. Therefore, there is a possibility that the present result obtained by using DSS-induced colitis model underestimated the actual value of KNT-127 expected in IBD. It may be constructive to further evaluate the effect of KNT-127 administration on colitis by using another colitis model, such as T cell transfer into T cell-deficient mice, which is an acquired immunity-dependent model.

In the current study, we found that significant changes in immune-related molecules and cells were caused by KNT-127 administration. The IL-6 concentration in the serum was decreased by KNT-127 administration, and in agreement, *in vitro* experiments revealed that KNT-127 inhibited the IL-6 and TNF-α production by LPS-stimulated macrophages. Moreover, the number of macrophages in the second lymphoid organs, one of the main sources of IL-6 (38, 39), was reduced in KNT-127-treated mice. Since the development and growth of BMDMs were not affected by KNT-127 (data not shown), the migratory ability of macrophages could be inhibited by KNT-127. The chemokine receptors involved in chemotaxis (50) are classified as G-protein coupled receptors (GPCRs), and all opioid receptors are GPCRs. GPCRs are known to be taken into the cytosol *via* endocytosis following by ligand binding (51, 52). Interestingly, the ligand-mediated GPCR internalization is caused by low specificity, and opioids and chemokines promote the intake of each other, resulting in signal desensitization (53). Thus, it is likely that KNT-127 reduces macrophage migration into the MLNs by modulating the function of chemokine receptors.

The presence of KNT-127 increased the population of Tregs *in vivo* and *in vitro*. KNT-127 strongly promoted naïve CD4^+^ T cell differentiation into Tregs in polarizing conditions *in vitro*. In addition, KNT-127 administration raised the population of Tregs in the spleen of healthy mice and in the MLNs of the colitis mice in the recovery phase, where are expected to be under Treg polarizing conditions; by contrast, the population was not altered in the colitis developing mice (**Supplementary Figure 5**), which is under inflammatory conditions. These observations may suggest that KNT-127 promotes differentiation toward the Treg phenotype under inducible conditions. Foxp3 is a transcription factor known to be a critical factor for the differentiation and maintenance of Tregs that is induced by TGF-β (54). Foxo1, which is a transcription factor involved in Foxp3 expression (55, 56), is reported to be activated by GPCR signaling (57, 58). Therefore, KNT-127 may facilitate the expression of Foxp3 *via* DOR in T cells. Epigenetic mechanisms are also crucial for the development of Tregs (59, 60). To clarify whether epigenetic regulations are involved in the acceleration of Treg development observed in the current study, the experiments investigating the degrees of histone acetylation and/or DNA methylation of KNT-127-treated Tregs may be useful.

In conclusion, we have shown that a DOR agonist, KNT-127, improves the pathology of DSS-induced colitis by regulating immune cells (**Figure 7** bottom). This study provides evidence that opioid drugs have the potential to be developed into immunomodulatory drugs. From the point of view of a translational medicine, we are going to perform further detailed analyses to clarify the molecular mechanisms by which opioid drugs modulate the function and development of immune cells and to evaluate the effects of opioid drugs on other immune-related diseases.

## Data Availability Statement

The original contributions presented in the study are included in the article/**Supplementary Material**. Further inquiries can be directed to the corresponding author.

## Ethics Statement

All animal experiments were performed in accordance with the guidelines of the Institutional Review Board of Tokyo University of Science. The current study was specifically approved by the Animal Care and Use Committees of Tokyo University of Science: K21004, K20005, K19006, K18006, K17009, and K17012.

## Author Contributions

KN performed experiments and analyzed data. KN and CN designed research and wrote the article. HN provided experimental materials and designed the research. AO performed preliminary experiments. AO and CN conceived the study. All authors contributed to the article and approved the submitted version.

## Funding

This work was supported by the Grant-in-Aid for Scientific Research (B) 20H02939 (CN), a Research Fellowship for Young Scientists DC2 and Grant-in-Aid for JSPS Fellows 21J12113 (KN), Tokyo University of Science Grant for President’s Research Promotion (CN), the MEXT-Supported Program for the Strategic Research Foundation at Private Universities (Translational Research Center, Tokyo University of Science), the Tojuro Iijima Foundation for Food Science and Technology (CN), a Research Grant from Takeda Science Foundation (CN), and a Research Grant from the Mishima Kaiun Memorial Foundation (CN).

## Conflict of Interest

The authors declare that the research was conducted in the absence of any commercial or financial relationships that could be construed as a potential conflict of interest.

## Publisher’s Note

All claims expressed in this article are solely those of the authors and do not necessarily represent those of their affiliated organizations, or those of the publisher, the editors and the reviewers. Any product that may be evaluated in this article, or claim that may be made by its manufacturer, is not guaranteed or endorsed by the publisher.

## Supplementary Material

Supplementary Figure 1 Effects of KNT-127 on a colitis recovery model. **(A)** Mice were given water containing 2.5% DSS to induce colitis from day 0 to day 8, and 5mg/kg KNT-127 (n=8) or vehicle (n=8) was administered intraperitoneally to the mice every 3 days from day -3 to day 9. Control mice (n=8) were given normal water. **(B)** The percentage of body weight to initial one. **(C)** Serum concentration of IL-6. **(D)** mRNA levels of Tnf and Il6 in colon. Symbol key; ○, control; □, vehicle; and △, KNT-127. The data are shown as the mean±s.e.m. **P* < 0.05; ***P* < 0.01; *****P* < 0.0001; n.s., not significant (one-way ANOVA followed by Dunnett’s multiple comparisons test, *vs* vehicle). i.p.; intraperitoneal.

Supplementary Figure 2 Changes in intestinal factors (colitis recovery model). The colon was collected from colitis mice on day 12. The mRNA expression of each gene of interest was normalized to that of GAPDH by calculation of cycle threshold values. **(A) ** mRNA levels of antimicrobial peptides. **(B)** mRNA levels of tight junction proteins. **(C)** mRNA levels of tissue restoration factors. The data are shown as the mean±s.e.m. ***P* < 0.01; ****P* < 0.001; *****P* < 0.0001; n.s., not significant (one-way ANOVA followed by Dunnett’s multiple comparisons test, *vs* vehicle).

Supplementary Figure 3 Changes in intestinal factors (control mice). The colon was collected from healthy mice 24 h after treatment with 5 mg/kg KNT-127 or vehicle. The mRNA expression of each gene of interest was normalized to that of GAPDH by calculation of cycle threshold values. **(A)** mRNA levels of antimicrobial peptides. **(B)** mRNA levels of tight junction proteins. **(C)** mRNA levels of tissue restoration factors. The data are shown as the mean±s.e.m. n.s., not significant (Two-tailed Student’s t-test).

Supplementary Figure 4 Frequency of CD4+ T cell subtypes in the MLNs (colitis recovery model). The MLNs were collected from colitis mice on day 12. The frequencies of IL-6^+^-, IFN-g^+^-, or IL-17A^+^-CD4^+^ T cells were determined by flow cytometry. **(A) ** Gating strategies. **(B)** Typical profiles of flow cytometry (top) and frequencies of cytokine-expressing cells (bottom; n = 3).

Supplementary Figure 5 Frequency of Tregs in the MLNs of the colitis developing mice. The MLNs were collected from colitis mice on day 8. Gating strategy was same as that in **Figure 4E**. **P* < 0.05; n.s., not significant (one-way ANOVA followed by Dunnett’s multiple comparisons test, *vs* vehicle).
