# Supplementary figures and images for: Delta Opioid Receptor Agonists Ameliorate Colonic Inflammation by Modulating Immune Responses
Source: Front Immunol. 2021 Sep 22;12:730706. doi: 10.3389/fimmu.2021.730706 (PMC8493000; doi:10.3389/fimmu.2021.730706)

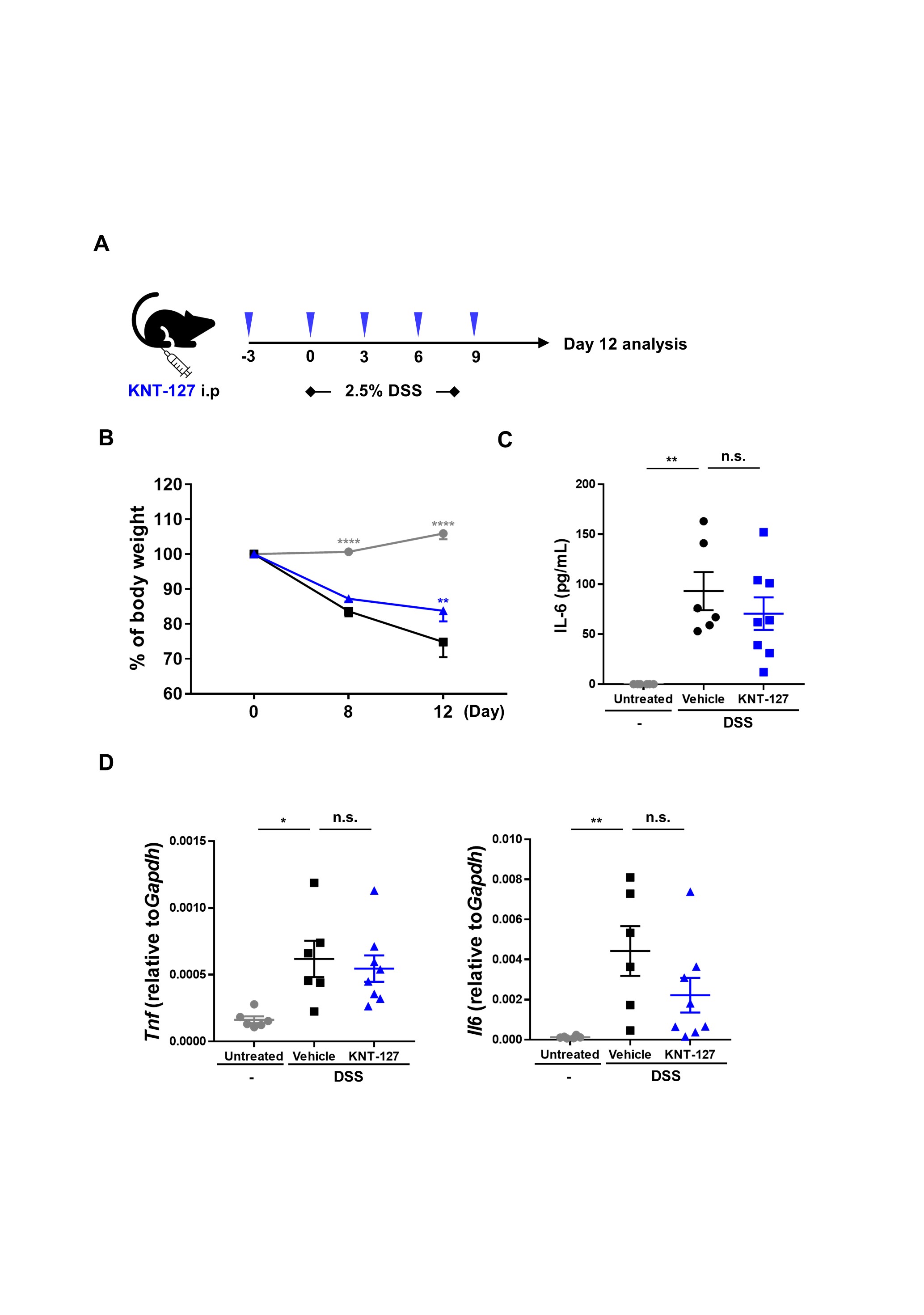

Supplement: Supplementary Figure 1 — Effects of KNT-127 on a colitis recovery model. (A) Mice were given water containing 2.5% DSS to induce colitis from day 0 to day 8, and 5mg/kg KNT-127 (n=8) or vehicle (n=8) was administered intraperitoneally to the mice every 3 days from day -3 to day 9. Control mice (n=8) were given normal water. (B) The percentage of body weight to initial one. (C) Serum concentration of IL-6. (D) mRNA levels of Tnf and Il6 in colon. Symbol key; ○, control; □, vehicle; and △, KNT-127. The data are shown as the mean±s.e.m. *P < 0.05; **P < 0.01; ****P < 0.0001; n.s., not significant (one-way ANOVA followed by Dunnett’s multiple comparisons test, vs vehicle). i.p.; intraperitoneal. [file Image_1.jpg]

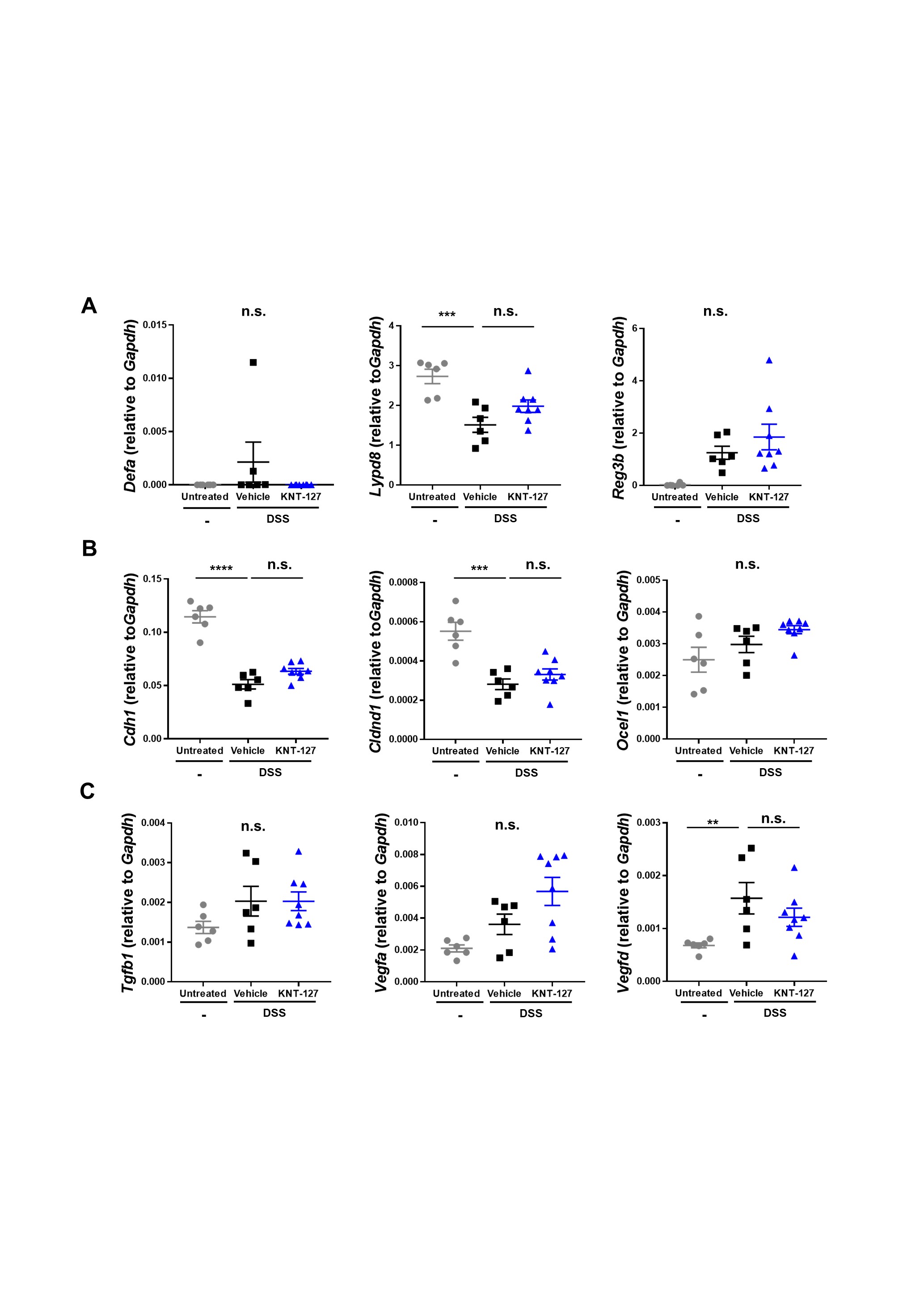

Supplement: Supplementary Figure 2 — Changes in intestinal factors (colitis recovery model). The colon was collected from colitis mice on day 12. The mRNA expression of each gene of interest was normalized to that of GAPDH by calculation of cycle threshold values. (A) mRNA levels of antimicrobial peptides. (B) mRNA levels of tight junction proteins. (C) mRNA levels of tissue restoration factors. The data are shown as the mean±s.e.m. **P < 0.01; ***P < 0.001; ****P < 0.0001; n.s., not significant (one-way ANOVA followed by Dunnett’s multiple comparisons test, vs vehicle). [file Image_2.jpg]

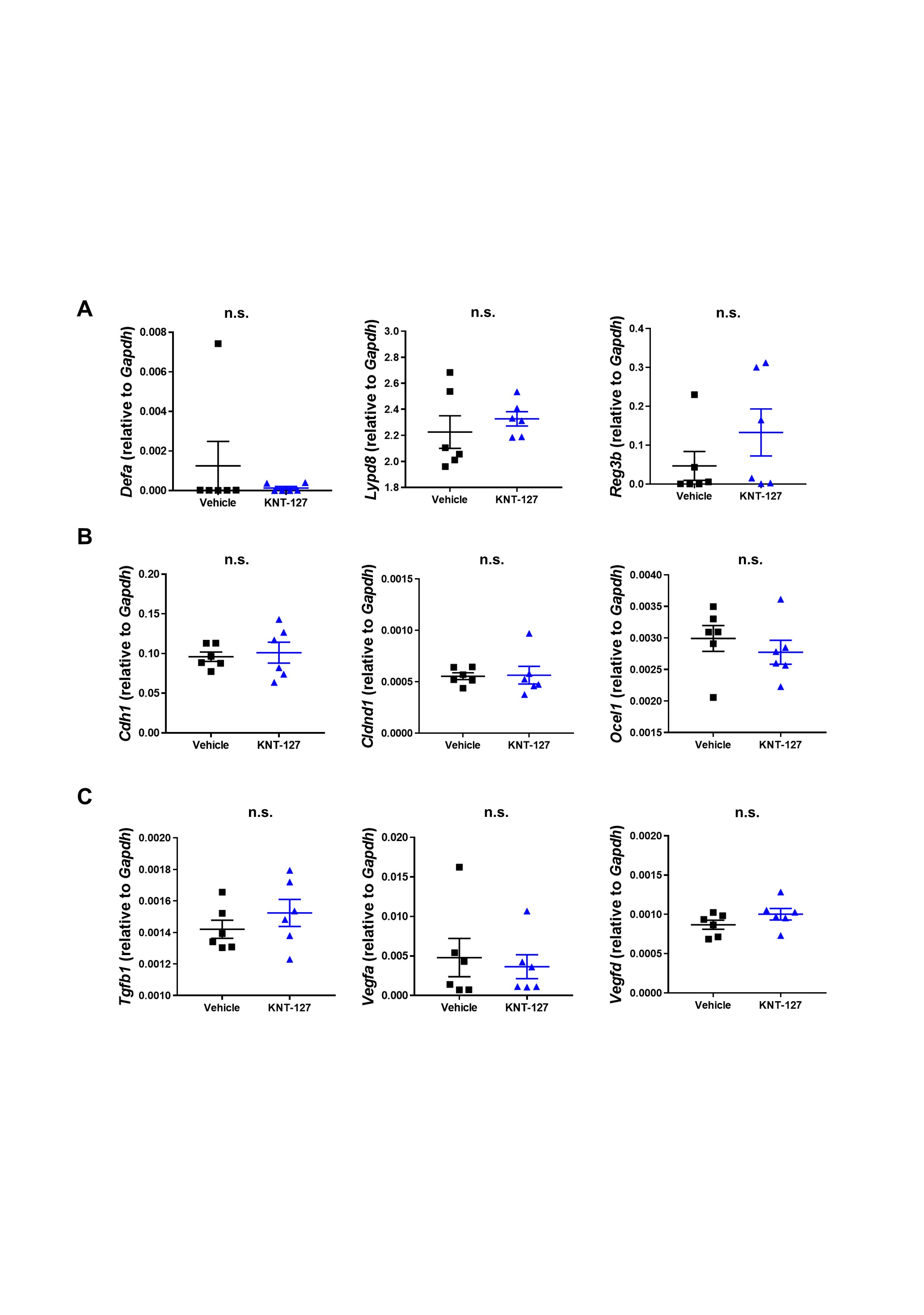

Supplement: Supplementary Figure 3 — Changes in intestinal factors (control mice). The colon was collected from healthy mice 24 h after treatment with 5 mg/kg KNT-127 or vehicle. The mRNA expression of each gene of interest was normalized to that of GAPDH by calculation of cycle threshold values. (A) mRNA levels of antimicrobial peptides. (B) mRNA levels of tight junction proteins. (C) mRNA levels of tissue restoration factors. The data are shown as the mean±s.e.m. n.s., not significant (Two-tailed Student’s t-test). [file Image_3.jpg]

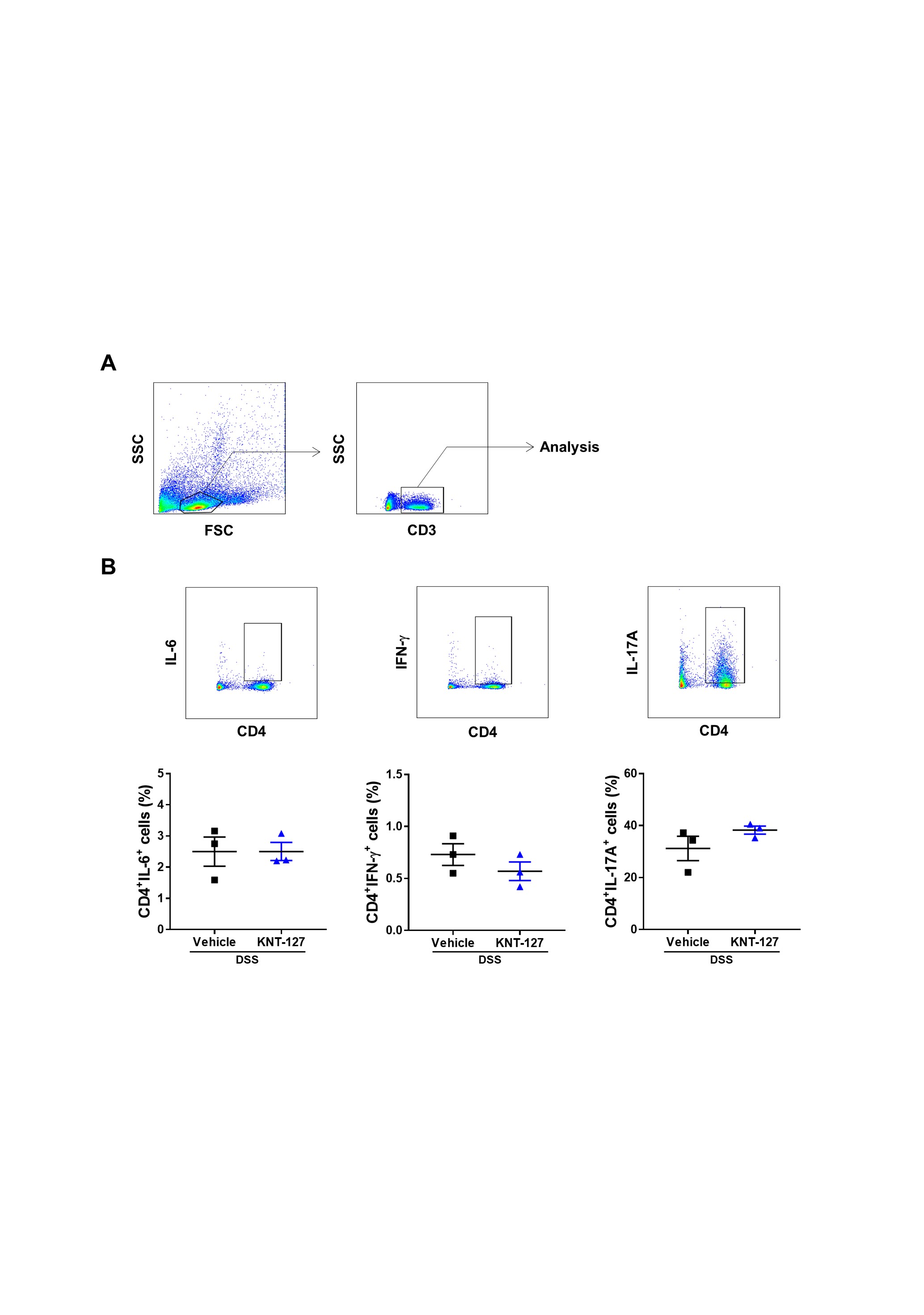

Supplement: Supplementary Figure 4 — Frequency of CD4+ T cell subtypes in the MLNs (colitis recovery model). The MLNs were collected from colitis mice on day 12. The frequencies of IL-6+-, IFN-g+-, or IL-17A+-CD4+ T cells were determined by flow cytometry. (A) Gating strategies. (B) Typical profiles of flow cytometry (top) and frequencies of cytokine-expressing cells (bottom; n = 3). [file Image_4.jpg]

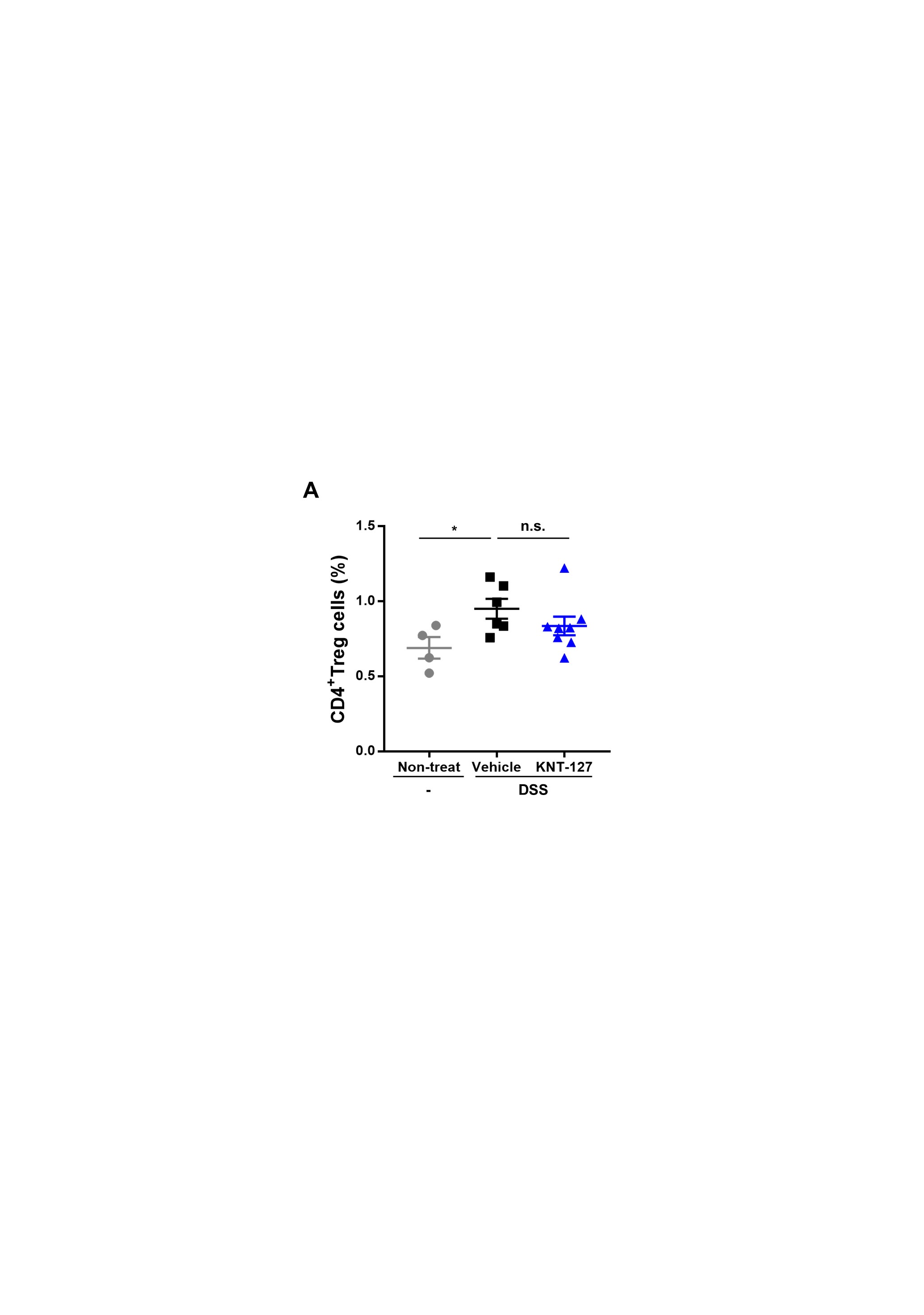

Supplement: Supplementary Figure 5 — Frequency of Tregs in the MLNs of the colitis developing mice. The MLNs were collected from colitis mice on day 8. Gating strategy was same as that in Figure 4E. *P < 0.05; n.s., not significant (one-way ANOVA followed by Dunnett’s multiple comparisons test, vs vehicle). [file Image_5.jpg]
